# 2-(5-Bromo-3-methyl­sulfanyl-1-benzofuran-2-yl)acetic acid

**DOI:** 10.1107/S1600536809005376

**Published:** 2009-02-21

**Authors:** Hong Dae Choi, Pil Ja Seo, Byeng Wha Son, Uk Lee

**Affiliations:** a Department of Chemistry, Dongeui University, San 24 Kaya-dong Busanjin-gu, Busan 614-714, Republic of Korea; b Department of Chemistry, Pukyong National University, 599-1 Daeyeon 3-dong Nam-gu, Busan 608-737, Republic of Korea

## Abstract

The title compound, C_11_H_9_BrO_3_S, was prepared by alkaline hydrolysis of ethyl 2-(5-bromo-3-methyl­sulfanyl-1-benzofuran-2-yl)acetate. In the crystal structure, the carboxyl groups are involved in inter­molecular O—H⋯O hydrogen bonds, which link the mol­ecules into centrosymmetric dimers. These dimers are further packed into stacks along the *c* axis by weak C—H⋯π inter­actions. In addition, the stacked mol­ecules exhibit a Br⋯S inter­action of 3.4787 (7) Å.

## Related literature

For the crystal structures of similar 2-(3-methyl­sulfanyl-1-benzofuran-2-yl)acetic acid derivatives, see: Choi *et al.* (2008*a* ▶,*b* ▶).
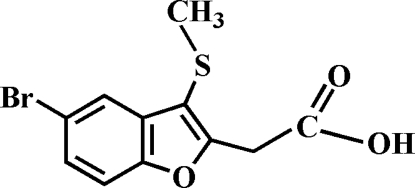


## Experimental

### 

#### Crystal data

C_11_H_9_BrO_3_S
*M* *_r_* = 301.15
Monoclinic, 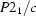

*a* = 4.9976 (4) Å
*b* = 29.740 (2) Å
*c* = 7.6780 (6) Å
β = 92.401 (1)°
*V* = 1140.17 (15) Å^3^
*Z* = 4
Mo *K*α radiation
μ = 3.78 mm^−1^
*T* = 100 K
0.50 × 0.30 × 0.15 mm

#### Data collection

Bruker SMART CCD diffractometer
Absorption correction: multi-scan (*SADABS*; Sheldrick, 2000 ▶) *T*_min_ = 0.261, *T*_max_ = 0.562
6872 measured reflections
2483 independent reflections
2178 reflections with *I* > 2σ(*I*)
*R*_int_ = 0.028

#### Refinement

*R*[*F* ^2^ > 2σ(*F* ^2^)] = 0.027
*wR*(*F* ^2^) = 0.072
*S* = 1.11
2483 reflections
150 parameters
H atoms treated by a mixture of independent and constrained refinement
Δρ_max_ = 0.32 e Å^−3^
Δρ_min_ = −0.56 e Å^−3^

### 

Data collection: *SMART* (Bruker, 2001 ▶); cell refinement: *SAINT* (Bruker, 2001 ▶); data reduction: *SAINT*; program(s) used to solve structure: *SHELXS97* (Sheldrick, 2008 ▶); program(s) used to refine structure: *SHELXL97* (Sheldrick, 2008 ▶); molecular graphics: *ORTEP-3* (Farrugia, 1997 ▶) and *DIAMOND* (Brandenburg, 1998 ▶); software used to prepare material for publication: *SHELXL97*.

## Supplementary Material

Crystal structure: contains datablocks global, I. DOI: 10.1107/S1600536809005376/fj2195sup1.cif

Structure factors: contains datablocks I. DOI: 10.1107/S1600536809005376/fj2195Isup2.hkl

Additional supplementary materials:  crystallographic information; 3D view; checkCIF report

## Figures and Tables

**Table 1:** Hydrogen-bond geometry (Å, °)

*D*—H⋯*A*	*D*—H	H⋯*A*	*D*⋯*A*	*D*—H⋯*A*
O3—H12⋯O2^i^	0.81 (4)	1.91 (4)	2.707 (2)	169 (4)
C11—H11*C*⋯*Cg*^ii^	0.96	3.22	3.904 (3)	129

Symmetry codes: (i) 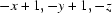; (ii) 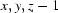. *Cg* is the centroid of the C2–C7 benzene ring.

**Fig. 1.**
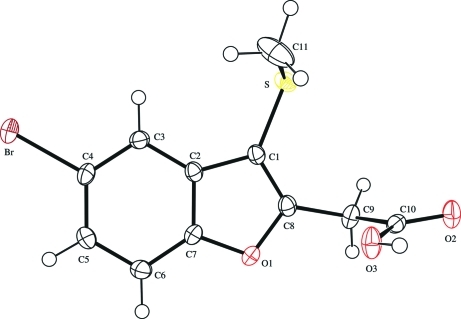
The molecular structure of the title compound, showing displacement ellipsoids drawn at the 50% probability level.

**Fig. 2.**
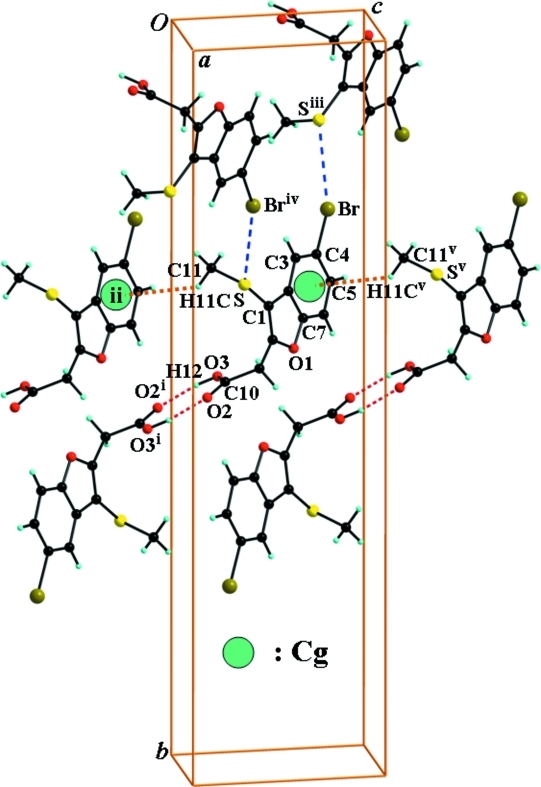
C—H···π and Br···S interactions, and hydrogen bonds (dotted lines) in the title compound. Cg denotes the ring centroid. [Symmetry code: (i) -x+1, -y+1, -z; (ii) x, y+1, z-1; (iii) x-1, -y+1/2, z+1/2; (iv) x+1, -y+1/2, z-1/2; (v) x, y, z+1.]

## supplementary crystallographic information

### Comment

As a part of our ongoing studies on the synthesis and structures of 2-(3-methylsulfanyl-1-benzofuran-2-yl)acetic acid analogues, the crystal structure of 2-(5,7-dimethyl-3-methylsulfanyl-1-benzofuran-2-yl)acetic acid (Choi *et al.*, 2008*a*) and 2-(6,7-dimethyl-3-methylsulfanyl-1-benzofuran-2-yl)acetic acid (Choi *et al.*, 2008*b*) have been described in the literature. Here we report the crystal structure of the title compound, 2-(5-bromo-3-methylsulfanyl-1-benzofuran-2-yl)acetic acid (Fig. 1).

The benzofuran unit is essentially planar, with a mean deviation of 0.011 (2) Å from the least-squares plane defined by the nine constituent atoms. In crystal structure, the carboxyl groups are involved in intermolecular O—H···O hydrogen bonds (Fig. 2 and Table 1; symmetry code as in Fig. 2), which link the molecules into centrosymmetric dimers. These dimers are further packed into stacks along the *c*–axis by weak C—H···π interactions, with a C11—H11C···*Cg*^ii^ separation of 3.22 Å (Fig. 2 and Table 1; *Cg* is the centroid of the C2–C7 benzene ring, symmetry code as in Fig. 2). Additionally, the stacked molecules exhibit a Br···S interaction, with a C4—Br···S^iii^ distance of 3.4787 (7) Å (symmetry code as in Fig. 2).

### Experimental

Ethyl 2-(5-bromo-3-methylsulfanyl-1-benzofuran-2-yl)acetate (329 mg, 1.0 mmol) was added to a solution of potassium hydroxide (337 mg, 6.0 mmol) in water (25 ml) and methanol (25 ml), and the mixture was refluxed for 5h, then cooled. Water was added, and the solution was extracted with dichloromethane. The aqueous layer was acidified to pH 1 with concentrated hydrochloric acid and then extracted with chloroform, dried over magnesium sulfate, filtered and concentrated under vacuum. The residue was purified by column chromatography (ethyl acetate) to afford the title compound as a colorless solid [yield 88%, m.p. 444-445 K; *R*_f_ = 0.56 (ethyl acetate)]. Single crystals suitable for X-ray diffraction were prepared by evaporation of a solution of the title compound in benzene at room temperature. Spectroscopic analysis: ^1^H NMR (CDCl_3_, 400 MHz) δ 2.32 (s, 3H), 4.04 (s, 2H), 7.33 (d, J = 8.8 Hz, 1H), 7.41 (dd, J = 8.8 Hz and J = 1.84 Hz, 1H), 7.77 (d, J = 2.2 Hz, 1H), 10.03 (s, 1H); EI-MS 302 [M+2], 300 [M^+^].

### Refinement

The H atom of O3 was positioned in a difference Fourier map and refined freely. Other H atoms were geometrically positioned and refined using a riding model, with C—H = 0.93 (aromatic), 0.97 (methylene), and 0.96 Å (methyl) H atoms, respectively, and with *U*_iso_(H) = 1.2Ueq(C) (aromatic, methylene), and 1.5Ueq(C) (methyl) H atoms.

### Crystal data

**Table:**

C_11_H_9_BrO_3_S	*F*(000) = 600
*M**_r_* = 301.15	*D*_x_ = 1.754 Mg m^−^^3^
Monoclinic, *P*2_1_/*c*	Melting point = 404–405 K
Hall symbol: -P 2ybc	Mo *K*α radiation, λ = 0.71073 Å
*a* = 4.9976 (4) Å	Cell parameters from 3612 reflections
*b* = 29.740 (2) Å	θ = 2.6–28.3°
*c* = 7.6780 (6) Å	µ = 3.78 mm^−^^1^
β = 92.401 (1)°	*T* = 100 K
*V* = 1140.17 (15) Å^3^	Block, colorless
*Z* = 4	0.50 × 0.30 × 0.15 mm

### Data collection

**Table:**

Bruker SMART CCD diffractometer	2483 independent reflections
Radiation source: fine-focus sealed tube	2178 reflections with *I* > 2σ(*I*)
graphite	*R*_int_ = 0.028
Detector resolution: 10.0 pixels mm^-1^	θ_max_ = 27.0°, θ_min_ = 1.4°
φ and ω scans	*h* = −6→6
Absorption correction: multi-scan (*SADABS*; Sheldrick, 2000)	*k* = −37→19
*T*_min_ = 0.261, *T*_max_ = 0.562	*l* = −9→9
6872 measured reflections	

### Refinement

**Table:**

Refinement on *F*^2^	Primary atom site location: structure-invariant direct methods
Least-squares matrix: full	Secondary atom site location: difference Fourier map
*R*[*F*^2^ > 2σ(*F*^2^)] = 0.027	Hydrogen site location: difference Fourier map
*wR*(*F*^2^) = 0.072	H atoms treated by a mixture of independent and constrained refinement
*S* = 1.11	*w* = 1/[σ^2^(*F*_o_^2^) + (0.0331*P*)^2^ + 0.6004*P*] where *P* = (*F*_o_^2^ + 2*F*_c_^2^)/3
2483 reflections	(Δ/σ)_max_ < 0.001
150 parameters	Δρ_max_ = 0.32 e Å^−^^3^
0 restraints	Δρ_min_ = −0.56 e Å^−^^3^

### Special details

**Table:**

Geometry. All esds (except the esd in the dihedral angle between two l.s. planes) are estimated using the full covariance matrix. The cell esds are taken into account individually in the estimation of esds in distances, angles and torsion angles; correlations between esds in cell parameters are only used when they are defined by crystal symmetry. An approximate (isotropic) treatment of cell esds is used for estimating esds involving l.s. planes.
Refinement. Refinement of F^2^ against ALL reflections. The weighted R-factor wR and goodness of fit S are based on F^2^, conventional R-factors R are based on F, with F set to zero for negative F^2^. The threshold expression of F^2^ > 2sigma(F^2^) is used only for calculating R-factors(gt) etc. and is not relevant to the choice of reflections for refinement. R-factors based on F^2^ are statistically about twice as large as those based on F, and R- factors based on ALL data will be even larger.

### Fractional atomic coordinates and isotropic or equivalent isotropic displacement parameters (Å^2^)

**Table:**

	*x*	*y*	*z*	*U*_iso_*/*U*_eq_	
Br	−0.17879 (5)	0.275363 (8)	0.83802 (3)	0.02932 (10)	
S	0.64485 (11)	0.33767 (2)	0.31284 (8)	0.02358 (14)	
O1	0.3933 (3)	0.43916 (5)	0.5972 (2)	0.0218 (3)	
O2	0.7696 (3)	0.48334 (6)	0.1003 (2)	0.0262 (4)	
O3	0.3598 (3)	0.47267 (7)	0.2002 (2)	0.0276 (4)	
H12	0.310 (7)	0.4829 (12)	0.107 (5)	0.057 (11)*	
C1	0.4952 (4)	0.37501 (8)	0.4556 (3)	0.0180 (4)	
C2	0.2974 (4)	0.36437 (8)	0.5820 (3)	0.0179 (4)	
C3	0.1697 (4)	0.32509 (8)	0.6316 (3)	0.0191 (5)	
H3	0.2048	0.2976	0.5797	0.023*	
C4	−0.0124 (5)	0.32894 (8)	0.7622 (3)	0.0200 (5)	
C5	−0.0721 (5)	0.36984 (8)	0.8405 (3)	0.0235 (5)	
H5	−0.1987	0.3708	0.9258	0.028*	
C6	0.0554 (5)	0.40921 (8)	0.7925 (3)	0.0234 (5)	
H6	0.0190	0.4368	0.8438	0.028*	
C7	0.2403 (4)	0.40497 (8)	0.6634 (3)	0.0190 (5)	
C8	0.5450 (4)	0.41944 (8)	0.4709 (3)	0.0198 (5)	
C9	0.7313 (5)	0.44956 (8)	0.3800 (3)	0.0226 (5)	
H9A	0.8911	0.4326	0.3554	0.027*	
H9B	0.7847	0.4737	0.4588	0.027*	
C10	0.6219 (4)	0.47002 (8)	0.2118 (3)	0.0192 (5)	
C11	0.3591 (6)	0.32394 (13)	0.1715 (4)	0.0455 (8)	
H11A	0.2212	0.3110	0.2391	0.068*	
H11B	0.4113	0.3027	0.0850	0.068*	
H11C	0.2925	0.3508	0.1152	0.068*	

### Atomic displacement parameters (Å^2^)

**Table:**

	*U*^11^	*U*^22^	*U*^33^	*U*^12^	*U*^13^	*U*^23^
Br	0.03467 (16)	0.02278 (15)	0.03138 (16)	−0.00670 (10)	0.01158 (10)	0.00524 (10)
S	0.0194 (3)	0.0267 (3)	0.0250 (3)	0.0028 (2)	0.0052 (2)	−0.0036 (3)
O1	0.0273 (8)	0.0150 (8)	0.0234 (8)	−0.0025 (7)	0.0048 (7)	0.0025 (7)
O2	0.0198 (8)	0.0332 (10)	0.0260 (9)	0.0008 (7)	0.0053 (7)	0.0115 (8)
O3	0.0169 (8)	0.0398 (11)	0.0262 (9)	−0.0008 (7)	0.0004 (7)	0.0135 (8)
C1	0.0180 (10)	0.0203 (11)	0.0159 (10)	0.0017 (9)	0.0022 (8)	0.0024 (9)
C2	0.0176 (10)	0.0206 (11)	0.0156 (10)	0.0014 (9)	0.0013 (8)	0.0023 (9)
C3	0.0234 (11)	0.0161 (11)	0.0178 (11)	−0.0001 (9)	0.0010 (9)	−0.0008 (9)
C4	0.0234 (11)	0.0186 (11)	0.0180 (11)	−0.0037 (9)	0.0007 (9)	0.0048 (9)
C5	0.0263 (12)	0.0265 (13)	0.0183 (11)	0.0000 (10)	0.0066 (9)	0.0001 (10)
C6	0.0294 (12)	0.0201 (12)	0.0210 (12)	0.0019 (10)	0.0043 (9)	−0.0021 (10)
C7	0.0208 (11)	0.0162 (11)	0.0199 (11)	−0.0012 (9)	0.0003 (9)	0.0037 (9)
C8	0.0194 (10)	0.0224 (12)	0.0176 (11)	0.0014 (9)	0.0010 (8)	0.0036 (9)
C9	0.0196 (11)	0.0232 (12)	0.0250 (12)	−0.0020 (9)	0.0003 (9)	0.0084 (10)
C10	0.0201 (11)	0.0149 (11)	0.0226 (11)	0.0002 (9)	0.0027 (9)	0.0017 (9)
C11	0.0288 (14)	0.073 (2)	0.0341 (15)	0.0025 (15)	−0.0009 (12)	−0.0262 (16)

### Geometric parameters (Å, °)

**Table:**

Br—C4	1.900 (2)	C3—H3	0.9300
Br—S^i^	3.4787 (7)	C4—C5	1.395 (3)
S—C1	1.750 (2)	C5—C6	1.390 (3)
S—C11	1.804 (3)	C5—H5	0.9300
O1—C7	1.382 (3)	C6—C7	1.389 (3)
O1—C8	1.386 (3)	C6—H6	0.9300
O2—C10	1.220 (3)	C8—C9	1.487 (3)
O3—C10	1.312 (3)	C9—C10	1.509 (3)
O3—H12	0.81 (4)	C9—H9A	0.9700
C1—C8	1.349 (3)	C9—H9B	0.9700
C1—C2	1.449 (3)	C11—H11A	0.9600
C2—C3	1.392 (3)	C11—H11B	0.9600
C2—C7	1.395 (3)	C11—H11C	0.9600
C3—C4	1.387 (3)		
			
C4—Br—S^i^	155.04 (7)	O1—C7—C6	126.2 (2)
C1—S—C11	99.90 (12)	O1—C7—C2	110.21 (19)
C7—O1—C8	105.87 (17)	C6—C7—C2	123.6 (2)
C10—O3—H12	111 (3)	C1—C8—O1	111.8 (2)
C8—C1—C2	106.5 (2)	C1—C8—C9	131.8 (2)
C8—C1—S	126.44 (18)	O1—C8—C9	116.4 (2)
C2—C1—S	127.04 (18)	C8—C9—C10	115.67 (19)
C3—C2—C7	120.0 (2)	C8—C9—H9A	108.4
C3—C2—C1	134.4 (2)	C10—C9—H9A	108.4
C7—C2—C1	105.6 (2)	C8—C9—H9B	108.4
C4—C3—C2	116.8 (2)	C10—C9—H9B	108.4
C4—C3—H3	121.6	H9A—C9—H9B	107.4
C2—C3—H3	121.6	O2—C10—O3	124.4 (2)
C3—C4—C5	122.8 (2)	O2—C10—C9	121.6 (2)
C3—C4—Br	117.46 (18)	O3—C10—C9	114.0 (2)
C5—C4—Br	119.68 (17)	S—C11—H11A	109.5
C6—C5—C4	120.8 (2)	S—C11—H11B	109.5
C6—C5—H5	119.6	H11A—C11—H11B	109.5
C4—C5—H5	119.6	S—C11—H11C	109.5
C7—C6—C5	116.0 (2)	H11A—C11—H11C	109.5
C7—C6—H6	122.0	H11B—C11—H11C	109.5
C5—C6—H6	122.0		
			
C11—S—C1—C8	−113.0 (2)	C5—C6—C7—O1	−178.9 (2)
C11—S—C1—C2	67.9 (2)	C5—C6—C7—C2	0.9 (4)
C8—C1—C2—C3	−178.7 (2)	C3—C2—C7—O1	178.60 (19)
S—C1—C2—C3	0.5 (4)	C1—C2—C7—O1	−1.1 (2)
C8—C1—C2—C7	0.9 (2)	C3—C2—C7—C6	−1.3 (4)
S—C1—C2—C7	−179.91 (17)	C1—C2—C7—C6	179.1 (2)
C7—C2—C3—C4	0.2 (3)	C2—C1—C8—O1	−0.4 (3)
C1—C2—C3—C4	179.8 (2)	S—C1—C8—O1	−179.63 (16)
C2—C3—C4—C5	1.1 (3)	C2—C1—C8—C9	178.6 (2)
C2—C3—C4—Br	−177.68 (16)	S—C1—C8—C9	−0.6 (4)
S^i^—Br—C4—C3	−12.5 (3)	C7—O1—C8—C1	−0.2 (2)
S^i^—Br—C4—C5	168.77 (12)	C7—O1—C8—C9	−179.39 (19)
C3—C4—C5—C6	−1.4 (4)	C1—C8—C9—C10	87.7 (3)
Br—C4—C5—C6	177.28 (18)	O1—C8—C9—C10	−93.3 (3)
C4—C5—C6—C7	0.4 (3)	C8—C9—C10—O2	−157.1 (2)
C8—O1—C7—C6	−179.3 (2)	C8—C9—C10—O3	24.1 (3)
C8—O1—C7—C2	0.8 (2)		

Symmetry codes: (i) *x*−1, −*y*+1/2, *z*+1/2.

### Hydrogen-bond geometry (Å, °)

**Table:**

*D*—H···*A*	*D*—H	H···*A*	*D*···*A*	*D*—H···*A*
O3—H12···O2^ii^	0.81 (4)	1.91 (4)	2.707 (2)	169 (4)
C11—H11C···Cg^iii^	0.96	3.22	3.904 (3)	129

Symmetry codes: (ii) −*x*+1, −*y*+1, −*z*; (iii) *x*, *y*, *z*−1.
